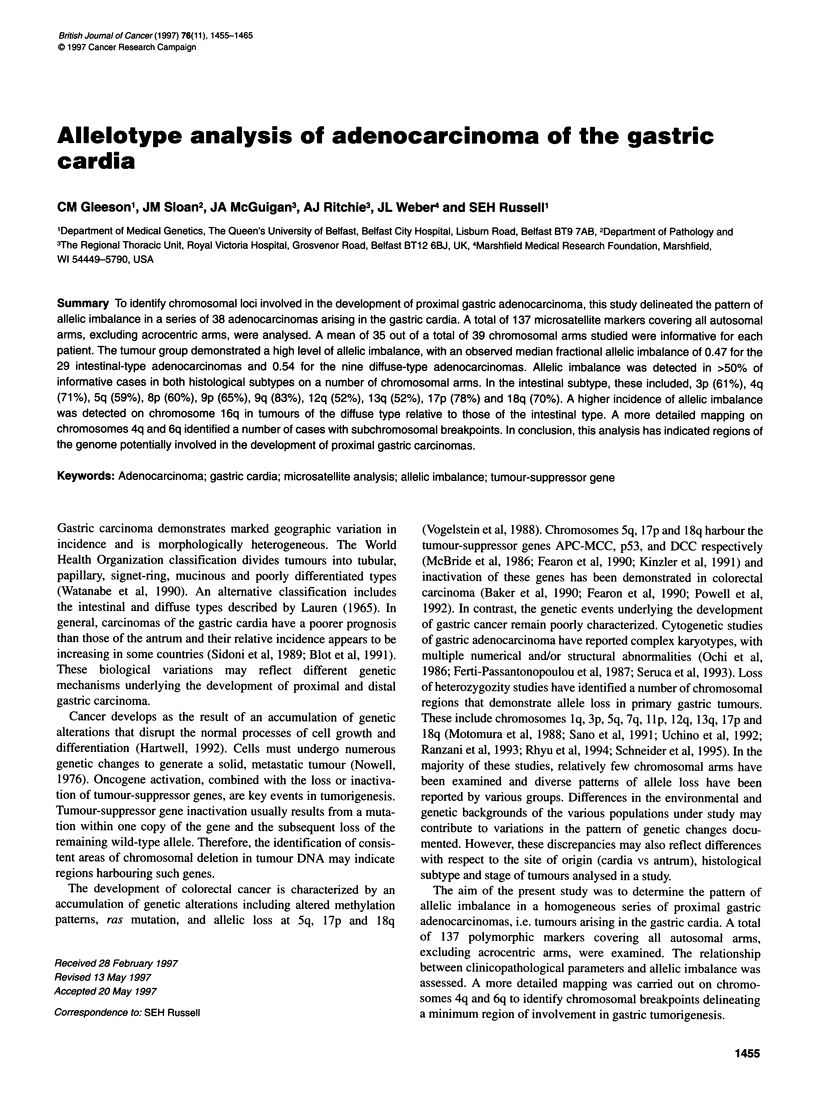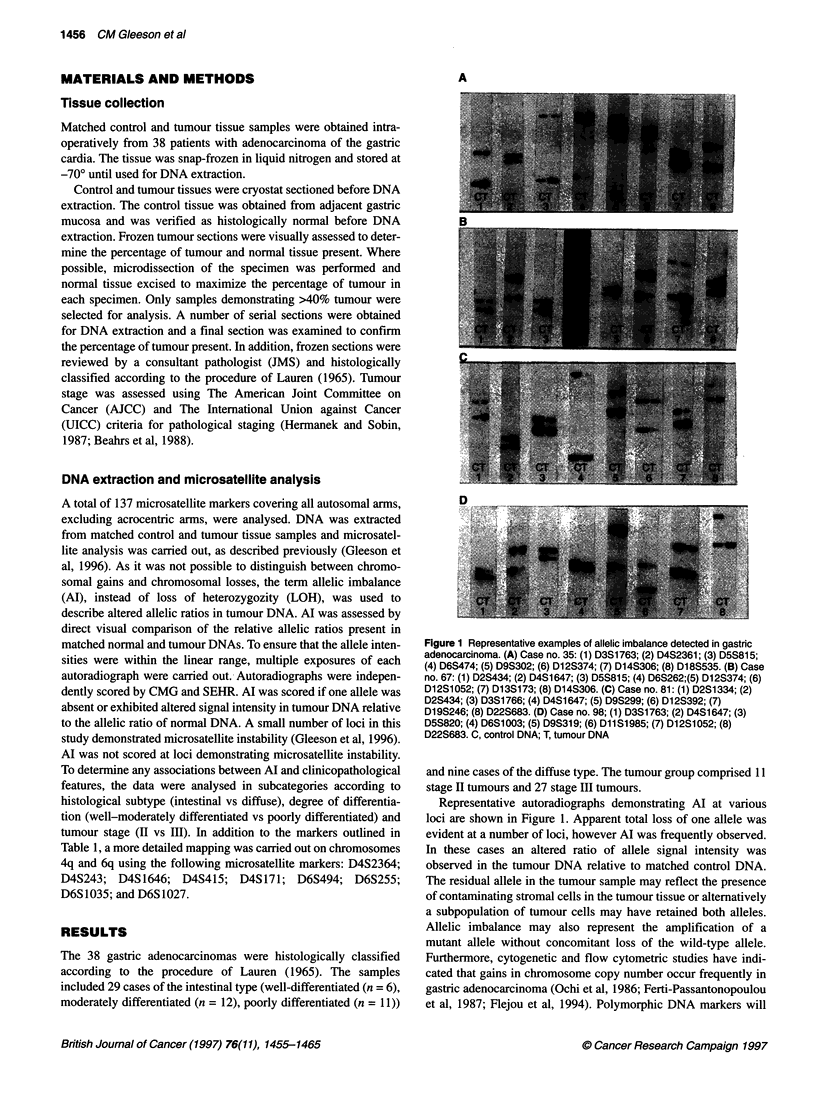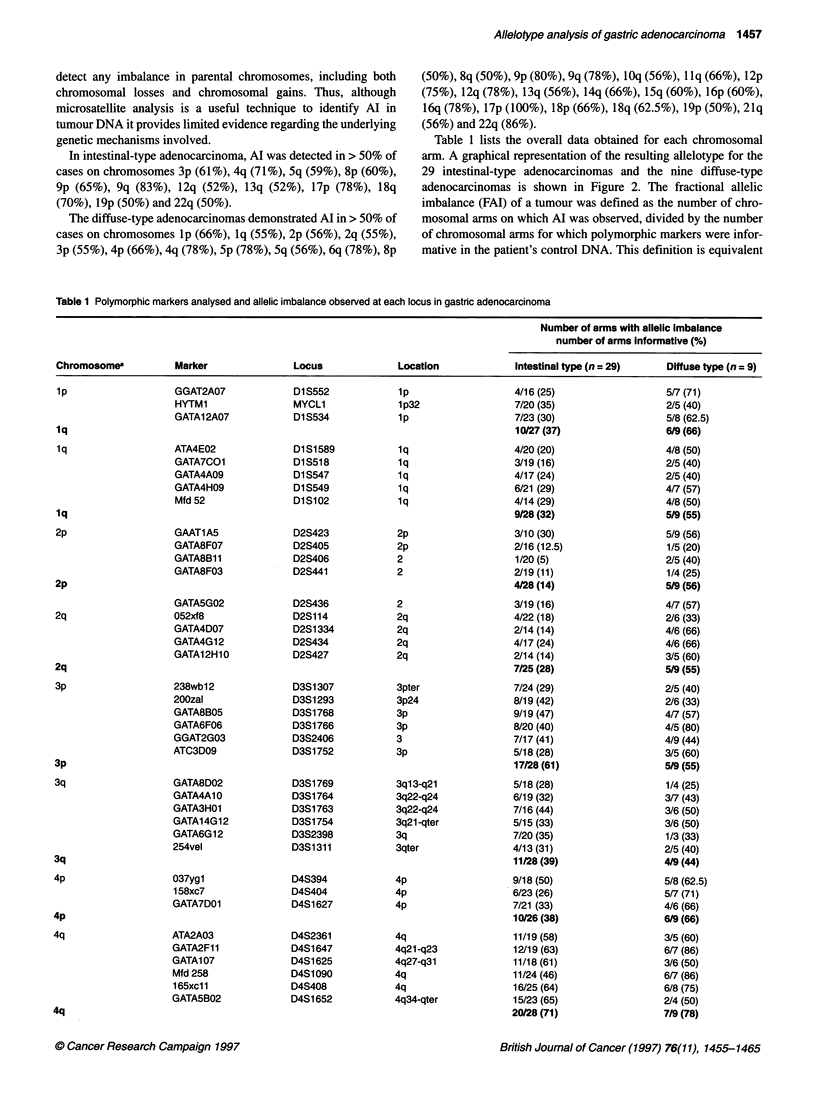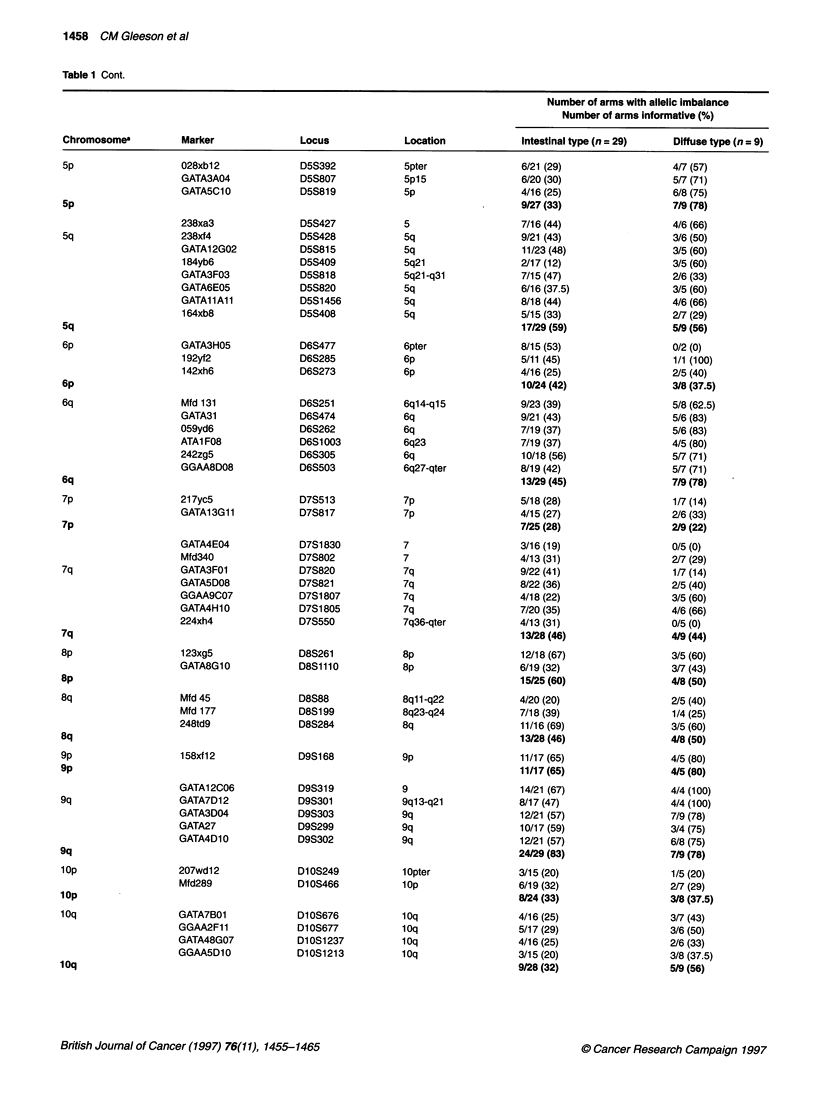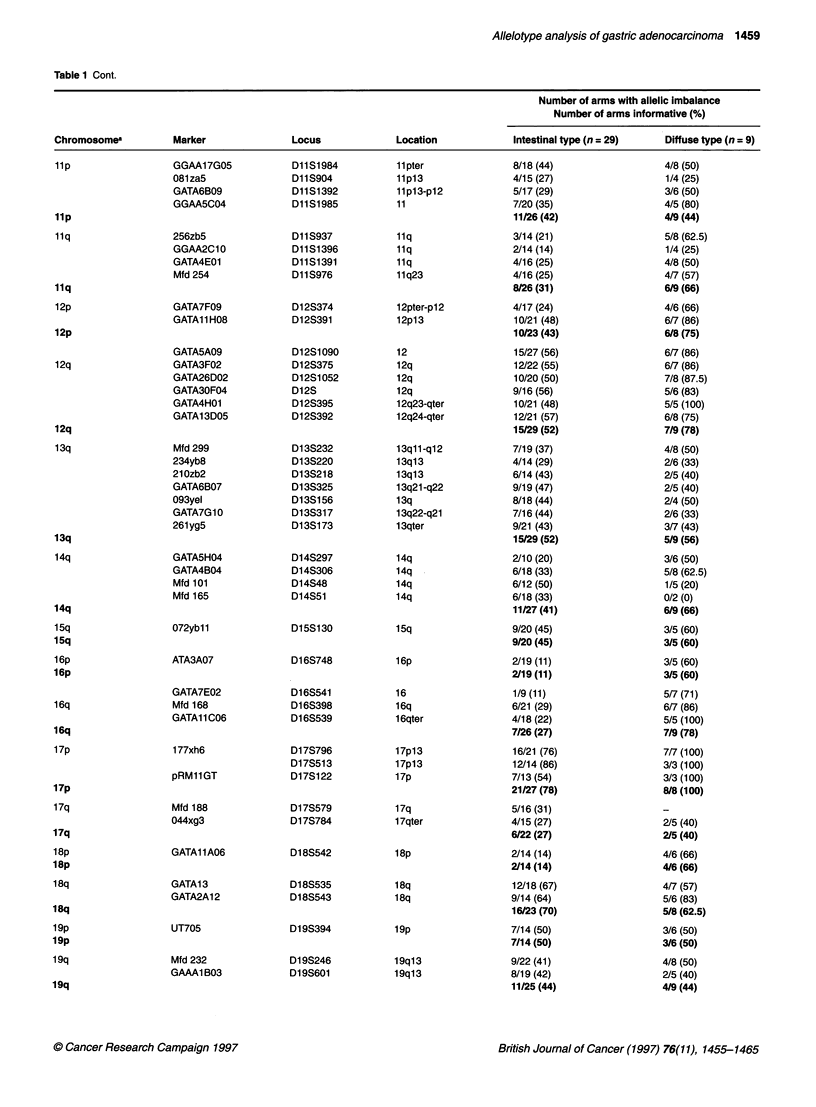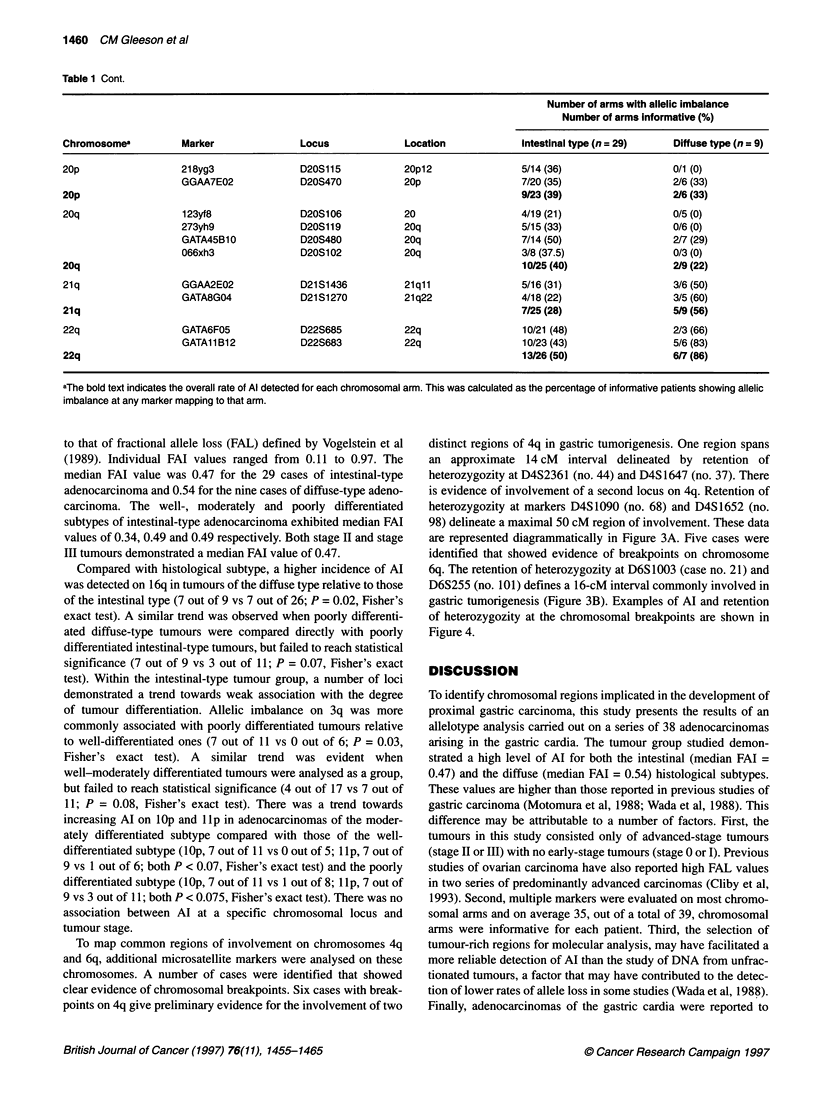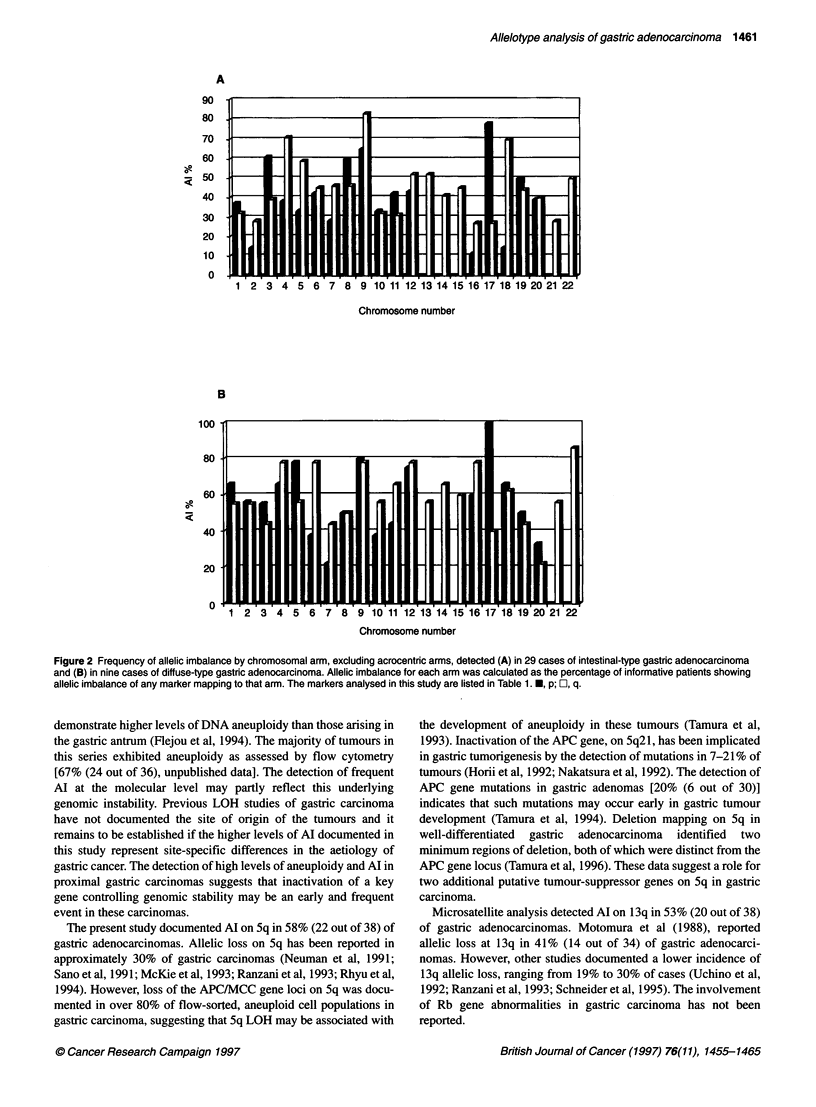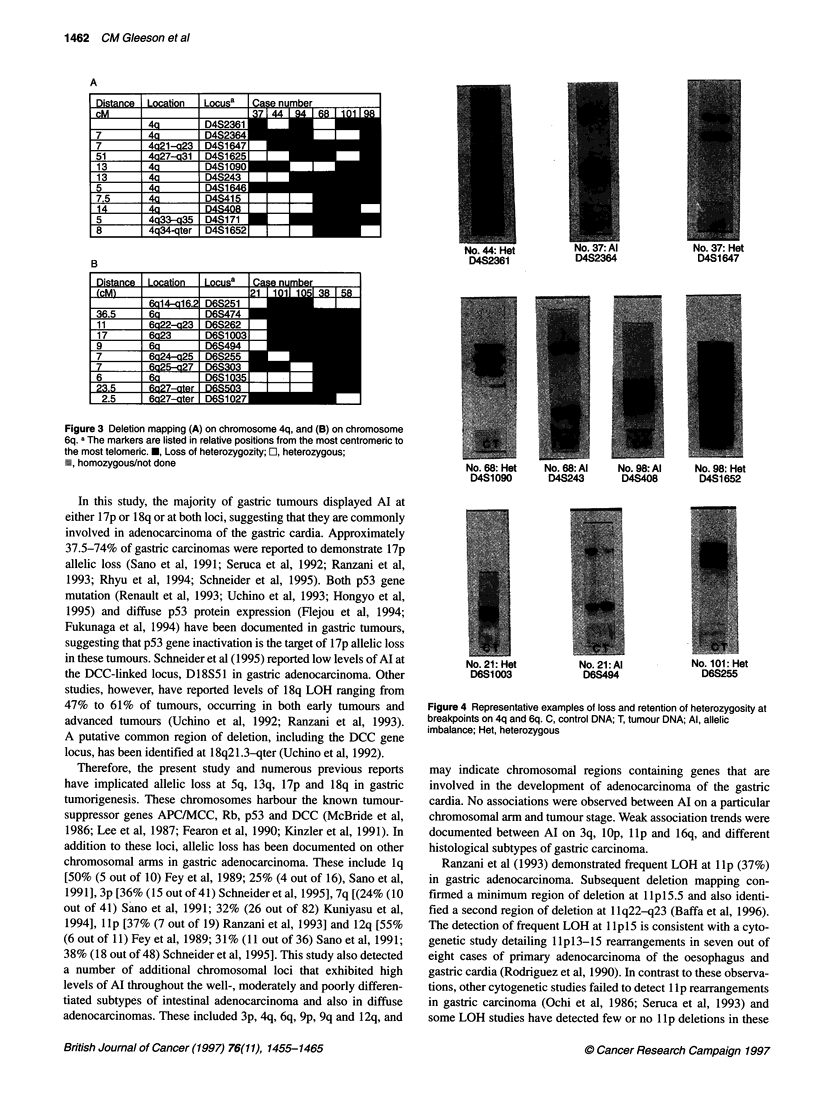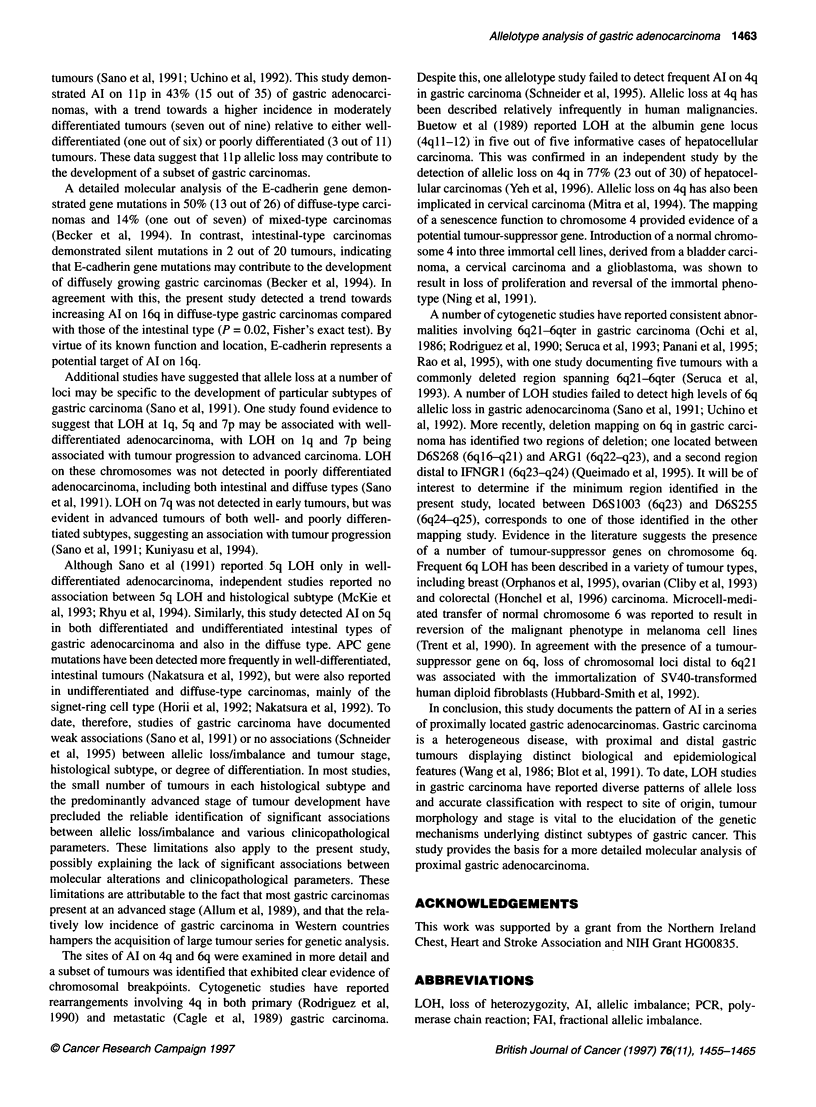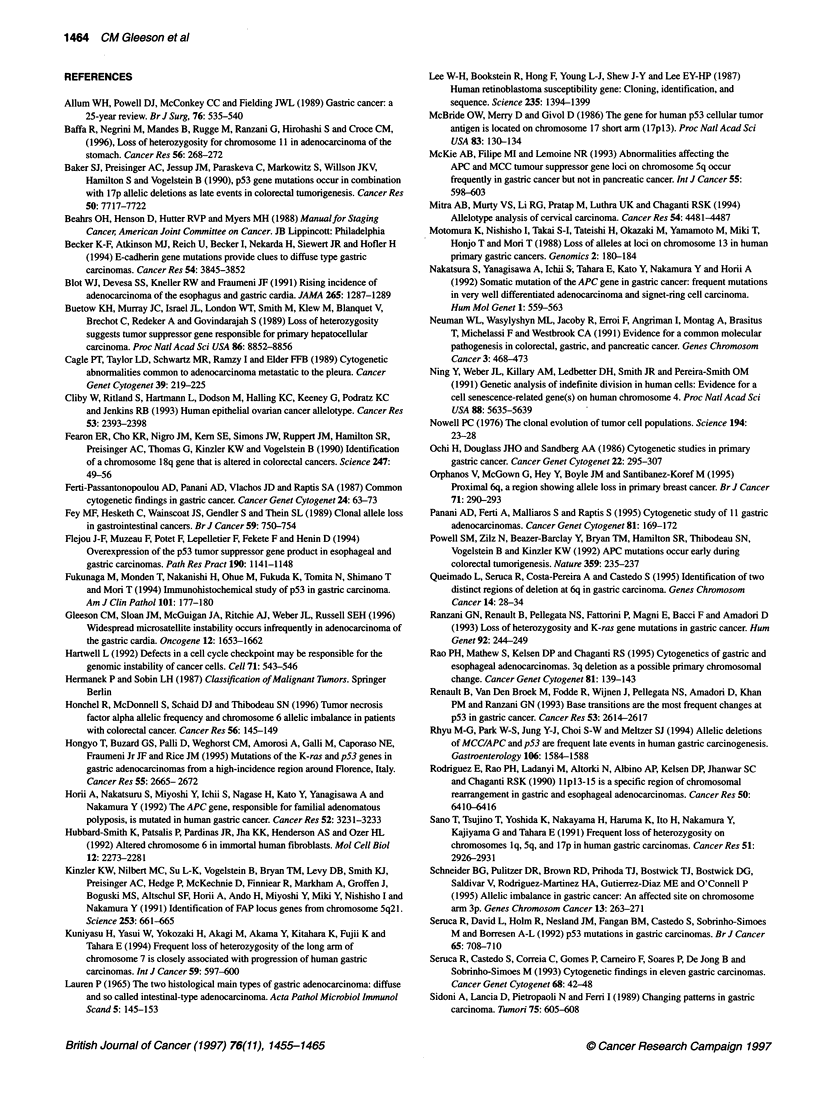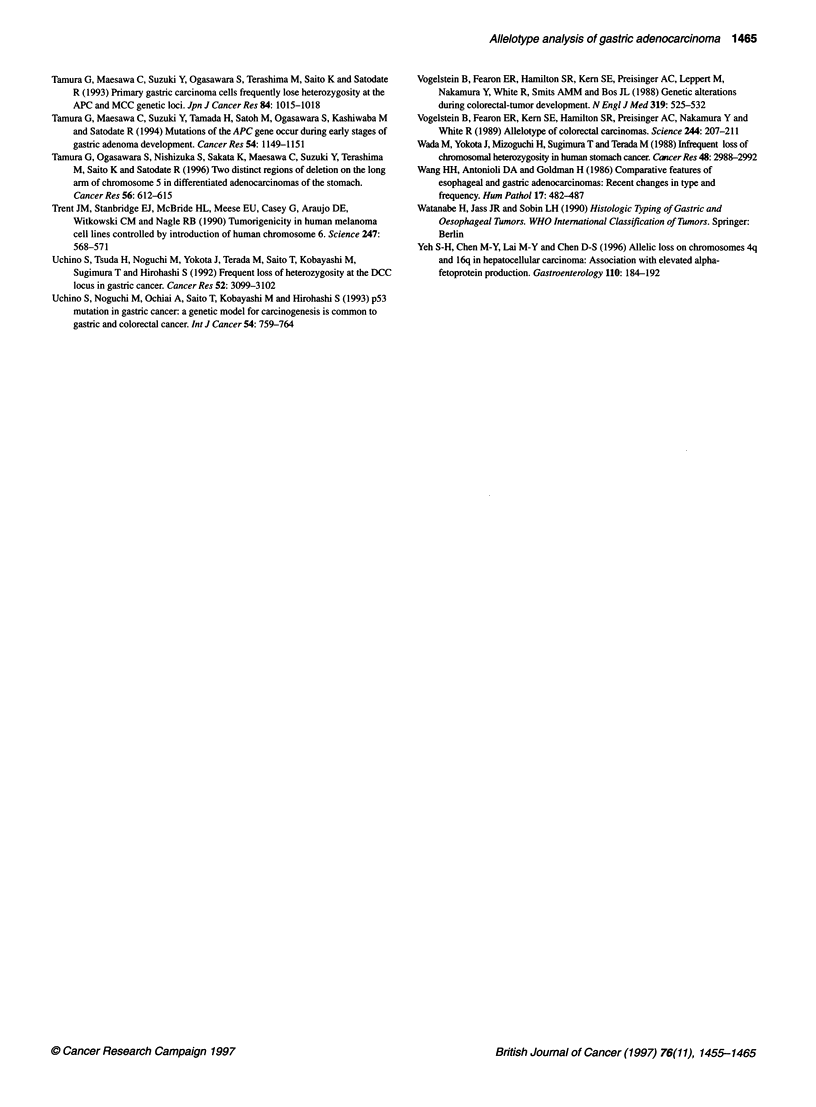# Allelotype analysis of adenocarcinoma of the gastric cardia.

**DOI:** 10.1038/bjc.1997.578

**Published:** 1997

**Authors:** C. M. Gleeson, J. M. Sloan, J. A. McGuigan, A. J. Ritchie, J. L. Weber, S. E. Russell

**Affiliations:** Department of Medical Genetics, The Queen's University of Belfast, Belfast City Hospital, UK.

## Abstract

**Images:**


British Journal of Cancer (1997) 76(11), 1455–1465
© 1997 Cancer Research Campaign

### Allelotype analysis of adenocarcinoma of the gastric cardia

**CM Gleeson[1], JM Sloan[2], JA McGuigan[3], AJ Ritchie[3], JL Weber[4] and SEH Russell[1]**

[1]Department of Medical Genetics, The Queen's University of Belfast, Belfast City Hospital, Lisburn Road, Belfast BT9 7AB, [2]Department of Pathology and [3]The Regional Thoracic Unit, Royal Victoria Hospital, Grosvenor Road, Belfast BT12 6BJ, UK, [4]Marshfield Medical Research Foundation, Marshfield, WI 54449–5790, USA

**Summary** To identify chromosomal loci involved in the development of proximal gastric adenocarcinoma, this study delineated the pattern of allelic imbalance in a series of 38 adenocarcinomas arising in the gastric cardia. A total of 137 microsatellite markers covering all autosomal arms, excluding acrocentric arms, were analysed. A mean of 35 out of a total of 39 chromosomal arms studied were informative for each patient. The tumour group demonstrated a high level of allelic imbalance, with an observed median fractional allelic imbalance of 0.47 for the 29 intestinal-type adenocarcinomas and 0.54 for the nine diffuse-type adenocarcinomas. Allelic imbalance was detected in >50% of informative cases in both histological subtypes on a number of chromosomal arms. In the intestinal subtype, these included, 3p (61%), 4q (71%), 5q (59%), 8p (60%), 9p (65%), 9q (83%), 12q (52%), 13q (52%), 17p (78%) and 18q (70%). A higher incidence of allelic imbalance was detected on chromosome 16q in tumours of the diffuse type relative to those of the intestinal type. A more detailed mapping on chromosomes 4q and 6q identified a number of cases with subchromosomal breakpoints. In conclusion, this analysis has indicated regions of the genome potentially involved in the development of proximal gastric carcinomas.

**Keywords:** Adenocarcinoma; gastric cardia; microsatellite analysis; allelic imbalance; tumour-suppressor gene

Gastric carcinoma demonstrates marked geographic variation in incidence and is morphologically heterogeneous. The World Health Organization classification divides tumours into tubular, papillary, signet-ring, mucinous and poorly differentiated types (Watanabe et al, 1990). An alternative classification includes the intestinal and diffuse types described by Lauren (1965). In general, carcinomas of the gastric cardia have a poorer prognosis than those of the antrum and their relative incidence appears to be increasing in some countries (Sidoni et al, 1989; Blot et al, 1991). These biological variations may reflect different genetic mechanisms underlying the development of proximal and distal gastric carcinoma.

Cancer develops as the result of an accumulation of genetic alterations that disrupt the normal processes of cell growth and differentiation (Hartwell, 1992). Cells must undergo numerous genetic changes to generate a solid, metastatic tumour (Nowell, 1976). Oncogene activation, combined with the loss or inactivation of tumour-suppressor genes, are key events in tumorigenesis. Tumour-suppressor gene inactivation usually results from a mutation within one copy of the gene and the subsequent loss of the remaining wild-type allele. Therefore, the identification of consistent areas of chromosomal deletion in tumour DNA may indicate regions harbouring such genes.

The development of colorectal cancer is characterized by an accumulation of genetic alterations including altered methylation patterns, *ras* mutation, and allelic loss at 5q, 17p and 18q (Vogelstein et al, 1988). Chromosomes 5q, 17p and 18q harbour the tumour-suppressor genes APC-MCC, p53, and DCC respectively (McBride et al, 1986; Fearon et al, 1990; Kinzler et al, 1991) and inactivation of these genes has been demonstrated in colorectal carcinoma (Baker et al, 1990; Fearon et al, 1990; Powell et al, 1992). In contrast, the genetic events underlying the development of gastric cancer remain poorly characterized. Cytogenetic studies of gastric adenocarcinoma have reported complex karyotypes, with multiple numerical and/or structural abnormalities (Ochi et al, 1986; Ferti-Passantonopoulou et al, 1987; Seruca et al, 1993). Loss of heterozygozity studies have identified a number of chromosomal regions that demonstrate allele loss in primary gastric tumours. These include chromosomes 1q, 3p, 5q, 7q, 11p, 12q, 13q, 17p and 18q (Motomura et al, 1988; Sano et al, 1991; Uchino et al, 1992; Ranzani et al, 1993; Rhyu et al, 1994; Schneider et al, 1995). In the majority of these studies, relatively few chromosomal arms have been examined and diverse patterns of allele loss have been reported by various groups. Differences in the environmental and genetic backgrounds of the various populations under study may contribute to variations in the pattern of genetic changes documented. However, these discrepancies may also reflect differences with respect to the site of origin (cardia vs antrum), histological subtype and stage of tumours analysed in a study.

The aim of the present study was to determine the pattern of allelic imbalance in a homogeneous series of proximal gastric adenocarcinomas, i.e. tumours arising in the gastric cardia. A total of 137 polymorphic markers covering all autosomal arms, excluding acrocentric arms, were examined. The relationship between clinicopathological parameters and allelic imbalance was assessed. A more detailed mapping was carried out on chromosomes 4q and 6q to identify chromosomal breakpoints delineating a minimum region of involvement in gastric tumorigenesis.

*Received 28 February 1997*
*Revised 13 May 1997*
*Accepted 20 May 1997*

*Correspondence to:* SEH Russell

### MATERIALS AND METHODS

### Tissue collection

Matched control and tumour tissue samples were obtained intraoperatively from 38 patients with adenocarcinoma of the gastric cardia. The tissue was snap-frozen in liquid nitrogen and stored at −70° until used for DNA extraction.

Control and tumour tissues were cryostat sectioned before DNA extraction. The control tissue was obtained from adjacent gastric mucosa and was verified as histologically normal before DNA extraction. Frozen tumour sections were visually assessed to determine the percentage of tumour and normal tissue present. Where possible, microdissection of the specimen was performed and normal tissue excised to maximize the percentage of tumour in each specimen. Only samples demonstrating >40% tumour were selected for analysis. A number of serial sections were obtained for DNA extraction and a final section was examined to confirm the percentage of tumour present. In addition, frozen sections were reviewed by a consultant pathologist (JMS) and histologically classified according to the procedure of Lauren (1965). Tumour stage was assessed using The American Joint Committee on Cancer (AJCC) and The International Union against Cancer (UICC) criteria for pathological staging (Hermanek and Sobin, 1987; Beahrs et al, 1988).

### DNA extraction and microsatellite analysis

A total of 137 microsatellite markers covering all autosomal arms, excluding acrocentric arms, were analysed. DNA was extracted from matched control and tumour tissue samples and microsatellite analysis was carried out, as described previously (Gleeson et al, 1996). As it was not possible to distinguish between chromosomal gains and chromosomal losses, the term allelic imbalance (AI), instead of loss of heterozygozity (LOH), was used to describe altered allelic ratios in tumour DNA. AI was assessed by direct visual comparison of the relative allelic ratios present in matched normal and tumour DNAs. To ensure that the allele intensities were within the linear range, multiple exposures of each autoradiograph were carried out. Autoradiographs were independently scored by CMG and SEHR. AI was scored if one allele was absent or exhibited altered signal intensity in tumour DNA relative to the allelic ratio of normal DNA. A small number of loci in this study demonstrated microsatellite instability (Gleeson et al, 1996). AI was not scored at loci demonstrating microsatellite instability. To determine any associations between AI and clinicopathological features, the data were analysed in subcategories according to histological subtype (intestinal vs diffuse), degree of differentiation (well–moderately differentiated vs poorly differentiated) and tumour stage (II vs III). In addition to the markers outlined in Table 1, a more detailed mapping was carried out on chromosomes 4q and 6q using the following microsatellite markers: D4S2364; D4S243; D4S1646; D4S415; D4S171; D6S494; D6S255; D6S1035; and D6S1027.

### RESULTS

The 38 gastric adenocarcinomas were histologically classified according to the procedure of Lauren (1965). The samples included 29 cases of the intestinal type (well-differentiated ($n$ = 6), moderately differentiated ($n$ = 12), poorly differentiated ($n$ = 11))

**Figure 1** Representative examples of allelic imbalance detected in gastric adenocarcinoma. **(A)** Case no. 35: (1) D3S1763; (2) D4S2361; (3) D5S815; (4) D6S474; (5) D9S302; (6) D12S374; (7) D14S306; (8) D18S535. **(B)** Case no. 67: (1) D2S434; (2) D4S1647; (3) D5S815; (4) D6S262;(5) D12S374; (6) D12S1052; (7) D13S173; (8) D14S306. **(C)** Case no. 81: (1) D2S1334; (2) D2S434; (3) D3S1766; (4) D4S1647; (5) D9S299; (6) D12S392; (7) D19S246; (8) D22S683. **(D)** Case no. 98; (1) D3S1763; (2) D4S1647; (3) D5S820; (4) D6S1003; (5) D9S319; (6) D11S1985; (7) D12S1052; (8) D22S683. C, control DNA; T, tumour DNA

and nine cases of the diffuse type. The tumour group comprised 11 stage II tumours and 27 stage III tumours.

Representative autoradiographs demonstrating AI at various loci are shown in Figure 1. Apparent total loss of one allele was evident at a number of loci, however AI was frequently observed. In these cases an altered ratio of allele signal intensity was observed in the tumour DNA relative to matched control DNA. The residual allele in the tumour sample may reflect the presence of contaminating stromal cells in the tumour tissue or alternatively a subpopulation of tumour cells may have retained both alleles. Allelic imbalance may also represent the amplification of a mutant allele without concomitant loss of the wild-type allele. Furthermore, cytogenetic and flow cytometric studies have indicated that gains in chromosome copy number occur frequently in gastric adenocarcinoma (Ochi et al, 1986; Ferti-Passantonopoulou et al, 1987; Flejou et al, 1994). Polymorphic DNA markers will

© Cancer Research Campaign 1997

detect any imbalance in parental chromosomes, including both chromosomal losses and chromosomal gains. Thus, although microsatellite analysis is a useful technique to identify AI in tumour DNA it provides limited evidence regarding the underlying genetic mechanisms involved.

In intestinal-type adenocarcinoma, AI was detected in > 50% of cases on chromosomes 3p (61%), 4q (71%), 5q (59%), 8p (60%), 9p (65%), 9q (83%), 12q (52%), 13q (52%), 17p (78%), 18q (70%), 19p (50%) and 22q (50%).

The diffuse-type adenocarcinomas demonstrated AI in > 50% of cases on chromosomes 1p (66%), 1q (55%), 2p (56%), 2q (55%), 3p (55%), 4p (66%), 4q (78%), 5p (78%), 5q (56%), 6q (78%), 8p (50%), 8q (50%), 9p (80%), 9q (78%), 10q (56%), 11q (66%), 12p (75%), 12q (78%), 13q (56%), 14q (66%), 15q (60%), 16p (60%), 16q (78%), 17p (100%), 18p (66%), 18q (62.5%), 19p (50%), 21q (56%) and 22q (86%).

Table 1 lists the overall data obtained for each chromosomal arm. A graphical representation of the resulting allelotype for the 29 intestinal-type adenocarcinomas and the nine diffuse-type adenocarcinomas is shown in Figure 2. The fractional allelic imbalance (FAI) of a tumour was defined as the number of chromosomal arms on which AI was observed, divided by the number of chromosomal arms for which polymorphic markers were informative in the patient's control DNA. This definition is equivalent

**Table 1** Polymorphic markers analysed and allelic imbalance observed at each locus in gastric adenocarcinoma

| | | | | Number of arms with allelic imbalance number of arms informative (%) | |
|---|---|---|---|---|---|
| **Chromosome[a]** | **Marker** | **Locus** | **Location** | **Intestinal type ($n$ = 29)** | **Diffuse type ($n$ = 9)** |
| 1p | GGAT2A07 | D1S552 | 1p | 4/16 (25) | 5/7 (71) |
| | HYTM1 | MYCL1 | 1p32 | 7/20 (35) | 2/5 (40) |
| | GATA12A07 | D1S534 | 1p | 7/23 (30) | 5/8 (62.5) |
| **1q** | | | | **10/27 (37)** | **6/9 (66)** |
| 1q | ATA4E02 | D1S1589 | 1q | 4/20 (20) | 4/8 (50) |
| | GATA7CO1 | D1S518 | 1q | 3/19 (16) | 2/5 (40) |
| | GATA4A09 | D1S547 | 1q | 4/17 (24) | 2/5 (40) |
| | GATA4H09 | D1S549 | 1q | 6/21 (29) | 4/7 (57) |
| | Mfd 52 | D1S102 | 1q | 4/14 (29) | 4/8 (50) |
| **1q** | | | | **9/28 (32)** | **5/9 (55)** |
| 2p | GAAT1A5 | D2S423 | 2p | 3/10 (30) | 5/9 (56) |
| | GATA8F07 | D2S405 | 2p | 2/16 (12.5) | 1/5 (20) |
| | GATA8B11 | D2S406 | 2 | 1/20 (5) | 2/5 (40) |
| | GATA8F03 | D2S441 | 2 | 2/19 (11) | 1/4 (25) |
| **2p** | | | | **4/28 (14)** | **5/9 (56)** |
| | GATA5G02 | D2S436 | 2 | 3/19 (16) | 4/7 (57) |
| 2q | 052xf8 | D2S114 | 2q | 4/22 (18) | 2/6 (33) |
| | GATA4D07 | D2S1334 | 2q | 2/14 (14) | 4/6 (66) |
| | GATA4G12 | D2S434 | 2q | 4/17 (24) | 4/6 (66) |
| | GATA12H10 | D2S427 | 2q | 2/14 (14) | 3/5 (60) |
| **2q** | | | | **7/25 (28)** | **5/9 (55)** |
| 3p | 238wb12 | D3S1307 | 3pter | 7/24 (29) | 2/5 (40) |
| | 200zal | D3S1293 | 3p24 | 8/19 (42) | 2/6 (33) |
| | GATA8B05 | D3S1768 | 3p | 9/19 (47) | 4/7 (57) |
| | GATA6F06 | D3S1766 | 3p | 8/20 (40) | 4/5 (80) |
| | GGAT2G03 | D3S2406 | 3 | 7/17 (41) | 4/9 (44) |
| | ATC3D09 | D3S1752 | 3p | 5/18 (28) | 3/5 (60) |
| **3p** | | | | **17/28 (61)** | **5/9 (55)** |
| 3q | GATA8D02 | D3S1769 | 3q13-q21 | 5/18 (28) | 1/4 (25) |
| | GATA4A10 | D3S1764 | 3q22-q24 | 6/19 (32) | 3/7 (43) |
| | GATA3H01 | D3S1763 | 3q22-q24 | 7/16 (44) | 3/6 (50) |
| | GATA14G12 | D3S1754 | 3q21-qter | 5/15 (33) | 3/6 (50) |
| | GATA6G12 | D3S2398 | 3q | 7/20 (35) | 1/3 (33) |
| | 254vel | D3S1311 | 3qter | 4/13 (31) | 2/5 (40) |
| **3q** | | | | **11/28 (39)** | **4/9 (44)** |
| 4p | 037yg1 | D4S394 | 4p | 9/18 (50) | 5/8 (62.5) |
| | 158xc7 | D4S404 | 4p | 6/23 (26) | 5/7 (71) |
| | GATA7D01 | D4S1627 | 4p | 7/21 (33) | 4/6 (66) |
| **4p** | | | | **10/26 (38)** | **6/9 (66)** |
| 4q | ATA2A03 | D4S2361 | 4q | 11/19 (58) | 3/5 (60) |
| | GATA2F11 | D4S1647 | 4q21-q23 | 12/19 (63) | 6/7 (86) |
| | GATA107 | D4S1625 | 4q27-q31 | 11/18 (61) | 3/6 (50) |
| | Mfd 258 | D4S1090 | 4q | 11/24 (46) | 6/7 (86) |
| | 165xc11 | D4S408 | 4q | 16/25 (64) | 6/8 (75) |
| | GATA5B02 | D4S1652 | 4q34-qter | 15/23 (65) | 2/4 (50) |
| **4q** | | | | **20/28 (71)** | **7/9 (78)** |

© Cancer Research Campaign 1997 

**Table 1** Cont.

| Chromosome[a] | Marker | Locus | Location | Number of arms with allelic imbalance / Number of arms informative (%) | |
|---|---|---|---|---|---|
| | | | | Intestinal type ($n$ = 29) | Diffuse type ($n$ = 9) |
| 5p | 028xb12 | D5S392 | 5pter | 6/21 (29) | 4/7 (57) |
| | GATA3A04 | D5S807 | 5p15 | 6/20 (30) | 5/7 (71) |
| | GATA5C10 | D5S819 | 5p | 4/16 (25) | 6/8 (75) |
| **5p** | | | | **9/27 (33)** | **7/9 (78)** |
| | 238xa3 | D5S427 | 5 | 7/16 (44) | 4/6 (66) |
| 5q | 238xf4 | D5S428 | 5q | 9/21 (43) | 3/6 (50) |
| | GATA12G02 | D5S815 | 5q | 11/23 (48) | 3/5 (60) |
| | 184yb6 | D5S409 | 5q21 | 2/17 (12) | 3/5 (60) |
| | GATA3F03 | D5S818 | 5q21-q31 | 7/15 (47) | 2/6 (33) |
| | GATA6E05 | D5S820 | 5q | 6/16 (37.5) | 3/5 (60) |
| | GATA11A11 | D5S1456 | 5q | 8/18 (44) | 4/6 (66) |
| | 164xb8 | D5S408 | 5q | 5/15 (33) | 2/7 (29) |
| **5q** | | | | **17/29 (59)** | **5/9 (56)** |
| 6p | GATA3H05 | D6S477 | 6pter | 8/15 (53) | 0/2 (0) |
| | 192yf2 | D6S285 | 6p | 5/11 (45) | 1/1 (100) |
| | 142xh6 | D6S273 | 6p | 4/16 (25) | 2/5 (40) |
| **6p** | | | | **10/24 (42)** | **3/8 (37.5)** |
| 6q | Mfd 131 | D6S251 | 6q14-q15 | 9/23 (39) | 5/8 (62.5) |
| | GATA31 | D6S474 | 6q | 9/21 (43) | 5/6 (83) |
| | 059yd6 | D6S262 | 6q | 7/19 (37) | 5/6 (83) |
| | ATA1F08 | D6S1003 | 6q23 | 7/19 (37) | 4/5 (80) |
| | 242zg5 | D6S305 | 6q | 10/18 (56) | 5/7 (71) |
| | GGAA8D08 | D6S503 | 6q27-qter | 8/19 (42) | 5/7 (71) |
| **6q** | | | | **13/29 (45)** | **7/9 (78)** |
| 7p | 217yc5 | D7S513 | 7p | 5/18 (28) | 1/7 (14) |
| | GATA13G11 | D7S817 | 7p | 4/15 (27) | 2/6 (33) |
| **7p** | | | | **7/25 (28)** | **2/9 (22)** |
| | GATA4E04 | D7S1830 | 7 | 3/16 (19) | 0/5 (0) |
| | Mfd340 | D7S802 | 7 | 4/13 (31) | 2/7 (29) |
| 7q | GATA3F01 | D7S820 | 7q | 9/22 (41) | 1/7 (14) |
| | GATA5D08 | D7S821 | 7q | 8/22 (36) | 2/5 (40) |
| | GGAA9C07 | D7S1807 | 7q | 4/18 (22) | 3/5 (60) |
| | GATA4H10 | D7S1805 | 7q | 7/20 (35) | 4/6 (66) |
| | 224xh4 | D7S550 | 7q36-qter | 4/13 (31) | 0/5 (0) |
| **7q** | | | | **13/28 (46)** | **4/9 (44)** |
| 8p | 123xg5 | D8S261 | 8p | 12/18 (67) | 3/5 (60) |
| | GATA8G10 | D8S1110 | 8p | 6/19 (32) | 3/7 (43) |
| **8p** | | | | **15/25 (60)** | **4/8 (50)** |
| 8q | Mfd 45 | D8S88 | 8q11-q22 | 4/20 (20) | 2/5 (40) |
| | Mfd 177 | D8S199 | 8q23-q24 | 7/18 (39) | 1/4 (25) |
| | 248td9 | D8S284 | 8q | 11/16 (69) | 3/5 (60) |
| **8q** | | | | **13/28 (46)** | **4/8 (50)** |
| 9p | 158xf12 | D9S168 | 9p | 11/17 (65) | 4/5 (80) |
| **9p** | | | | **11/17 (65)** | **4/5 (80)** |
| | GATA12C06 | D9S319 | 9 | 14/21 (67) | 4/4 (100) |
| 9q | GATA7D12 | D9S301 | 9q13-q21 | 8/17 (47) | 4/4 (100) |
| | GATA3D04 | D9S303 | 9q | 12/21 (57) | 7/9 (78) |
| | GATA27 | D9S299 | 9q | 10/17 (59) | 3/4 (75) |
| | GATA4D10 | D9S302 | 9q | 12/21 (57) | 6/8 (75) |
| **9q** | | | | **24/29 (83)** | **7/9 (78)** |
| 10p | 207wd12 | D10S249 | 10pter | 3/15 (20) | 1/5 (20) |
| | Mfd289 | D10S466 | 10p | 6/19 (32) | 2/7 (29) |
| **10p** | | | | **8/24 (33)** | **3/8 (37.5)** |
| 10q | GATA7B01 | D10S676 | 10q | 4/16 (25) | 3/7 (43) |
| | GGAA2F11 | D10S677 | 10q | 5/17 (29) | 3/6 (50) |
| | GATA48G07 | D10S1237 | 10q | 4/16 (25) | 2/6 (33) |
| | GGAA5D10 | D10S1213 | 10q | 3/15 (20) | 3/8 (37.5) |
| **10q** | | | | **9/28 (32)** | **5/9 (56)** |

*© Cancer Research Campaign 1997*

**Table 1** Cont.

| Chromosome[a] | Marker | Locus | Location | Number of arms with allelic imbalance / Number of arms informative (%) Intestinal type (*n* = 29) | Diffuse type (*n* = 9) |
|---|---|---|---|---|---|
| 11p | GGAA17G05 | D11S1984 | 11pter | 8/18 (44) | 4/8 (50) |
| | 081za5 | D11S904 | 11p13 | 4/15 (27) | 1/4 (25) |
| | GATA6B09 | D11S1392 | 11p13-p12 | 5/17 (29) | 3/6 (50) |
| | GGAA5C04 | D11S1985 | 11 | 7/20 (35) | 4/5 (80) |
| **11p** | | | | **11/26 (42)** | **4/9 (44)** |
| 11q | 256zb5 | D11S937 | 11q | 3/14 (21) | 5/8 (62.5) |
| | GGAA2C10 | D11S1396 | 11q | 2/14 (14) | 1/4 (25) |
| | GATA4E01 | D11S1391 | 11q | 4/16 (25) | 4/8 (50) |
| | Mfd 254 | D11S976 | 11q23 | 4/16 (25) | 4/7 (57) |
| **11q** | | | | **8/26 (31)** | **6/9 (66)** |
| 12p | GATA7F09 | D12S374 | 12pter-p12 | 4/17 (24) | 4/6 (66) |
| | GATA11H08 | D12S391 | 12p13 | 10/21 (48) | 6/7 (86) |
| **12p** | | | | **10/23 (43)** | **6/8 (75)** |
| | GATA5A09 | D12S1090 | 12 | 15/27 (56) | 6/7 (86) |
| 12q | GATA3F02 | D12S375 | 12q | 12/22 (55) | 6/7 (86) |
| | GATA26D02 | D12S1052 | 12q | 10/20 (50) | 7/8 (87.5) |
| | GATA30F04 | D12S | 12q | 9/16 (56) | 5/6 (83) |
| | GATA4H01 | D12S395 | 12q23-qter | 10/21 (48) | 5/5 (100) |
| | GATA13D05 | D12S392 | 12q24-qter | 12/21 (57) | 6/8 (75) |
| **12q** | | | | **15/29 (52)** | **7/9 (78)** |
| 13q | Mfd 299 | D13S232 | 13q11-q12 | 7/19 (37) | 4/8 (50) |
| | 234yb8 | D13S220 | 13q13 | 4/14 (29) | 2/6 (33) |
| | 210zb2 | D13S218 | 13q13 | 6/14 (43) | 2/5 (40) |
| | GATA6B07 | D13S325 | 13q21-q22 | 9/19 (47) | 2/5 (40) |
| | 093yel | D13S156 | 13q | 8/18 (44) | 2/4 (50) |
| | GATA7G10 | D13S317 | 13q22-q21 | 7/16 (44) | 2/6 (33) |
| | 261yg5 | D13S173 | 13qter | 9/21 (43) | 3/7 (43) |
| **13q** | | | | **15/29 (52)** | **5/9 (56)** |
| 14q | GATA5H04 | D14S297 | 14q | 2/10 (20) | 3/6 (50) |
| | GATA4B04 | D14S306 | 14q | 6/18 (33) | 5/8 (62.5) |
| | Mfd 101 | D14S48 | 14q | 6/12 (50) | 1/5 (20) |
| | Mfd 165 | D14S51 | 14q | 6/18 (33) | 0/2 (0) |
| **14q** | | | | **11/27 (41)** | **6/9 (66)** |
| 15q | 072yb11 | D15S130 | 15q | 9/20 (45) | 3/5 (60) |
| **15q** | | | | **9/20 (45)** | **3/5 (60)** |
| 16p | ATA3A07 | D16S748 | 16p | 2/19 (11) | 3/5 (60) |
| **16p** | | | | **2/19 (11)** | **3/5 (60)** |
| | GATA7E02 | D16S541 | 16 | 1/9 (11) | 5/7 (71) |
| 16q | Mfd 168 | D16S398 | 16q | 6/21 (29) | 6/7 (86) |
| | GATA11C06 | D16S539 | 16qter | 4/18 (22) | 5/5 (100) |
| **16q** | | | | **7/26 (27)** | **7/9 (78)** |
| 17p | 177xh6 | D17S796 | 17p13 | 16/21 (76) | 7/7 (100) |
| | | D17S513 | 17p13 | 12/14 (86) | 3/3 (100) |
| | pRM11GT | D17S122 | 17p | 7/13 (54) | 3/3 (100) |
| **17p** | | | | **21/27 (78)** | **8/8 (100)** |
| 17q | Mfd 188 | D17S579 | 17q | 5/16 (31) | – |
| | 044xg3 | D17S784 | 17qter | 4/15 (27) | 2/5 (40) |
| **17q** | | | | **6/22 (27)** | **2/5 (40)** |
| 18p | GATA11A06 | D18S542 | 18p | 2/14 (14) | 4/6 (66) |
| **18p** | | | | **2/14 (14)** | **4/6 (66)** |
| 18q | GATA13 | D18S535 | 18q | 12/18 (67) | 4/7 (57) |
| | GATA2A12 | D18S543 | 18q | 9/14 (64) | 5/6 (83) |
| **18q** | | | | **16/23 (70)** | **5/8 (62.5)** |
| 19p | UT705 | D19S394 | 19p | 7/14 (50) | 3/6 (50) |
| **19p** | | | | **7/14 (50)** | **3/6 (50)** |
| 19q | Mfd 232 | D19S246 | 19q13 | 9/22 (41) | 4/8 (50) |
| | GAAA1B03 | D19S601 | 19q13 | 8/19 (42) | 2/5 (40) |
| **19q** | | | | **11/25 (44)** | **4/9 (44)** |

© Cancer Research Campaign 1997

Table 1 Cont.

| Chromosome[a] | Marker | Locus | Location | Number of arms with allelic imbalance / Number of arms informative (%) | |
|---|---|---|---|---|---|
| | | | | Intestinal type ($n$ = 29) | Diffuse type ($n$ = 9) |
| 20p | 218yg3 | D20S115 | 20p12 | 5/14 (36) | 0/1 (0) |
| | GGAA7E02 | D20S470 | 20p | 7/20 (35) | 2/6 (33) |
| **20p** | | | | **9/23 (39)** | **2/6 (33)** |
| 20q | 123yf8 | D20S106 | 20 | 4/19 (21) | 0/5 (0) |
| | 273yh9 | D20S119 | 20q | 5/15 (33) | 0/6 (0) |
| | GATA45B10 | D20S480 | 20q | 7/14 (50) | 2/7 (29) |
| | 066xh3 | D20S102 | 20q | 3/8 (37.5) | 0/3 (0) |
| **20q** | | | | **10/25 (40)** | **2/9 (22)** |
| 21q | GGAA2E02 | D21S1436 | 21q11 | 5/16 (31) | 3/6 (50) |
| | GATA8G04 | D21S1270 | 21q22 | 4/18 (22) | 3/5 (60) |
| **21q** | | | | **7/25 (28)** | **5/9 (56)** |
| 22q | GATA6F05 | D22S685 | 22q | 10/21 (48) | 2/3 (66) |
| | GATA11B12 | D22S683 | 22q | 10/23 (43) | 5/6 (83) |
| **22q** | | | | **13/26 (50)** | **6/7 (86)** |

[a]The bold text indicates the overall rate of AI detected for each chromosomal arm. This was calculated as the percentage of informative patients showing allelic imbalance at any marker mapping to that arm.

to that of fractional allele loss (FAL) defined by Vogelstein et al (1989). Individual FAI values ranged from 0.11 to 0.97. The median FAI value was 0.47 for the 29 cases of intestinal-type adenocarcinoma and 0.54 for the nine cases of diffuse-type adenocarcinoma. The well-, moderately and poorly differentiated subtypes of intestinal-type adenocarcinoma exhibited median FAI values of 0.34, 0.49 and 0.49 respectively. Both stage II and stage III tumours demonstrated a median FAI value of 0.47.

Compared with histological subtype, a higher incidence of AI was detected on 16q in tumours of the diffuse type relative to those of the intestinal type (7 out of 9 vs 7 out of 26; $P = 0.02$, Fisher's exact test). A similar trend was observed when poorly differentiated diffuse-type tumours were compared directly with poorly differentiated intestinal-type tumours, but failed to reach statistical significance (7 out of 9 vs 3 out of 11; $P = 0.07$, Fisher's exact test). Within the intestinal-type tumour group, a number of loci demonstrated a trend towards weak association with the degree of tumour differentiation. Allelic imbalance on 3q was more commonly associated with poorly differentiated tumours relative to well-differentiated ones (7 out of 11 vs 0 out of 6; $P = 0.03$, Fisher's exact test). A similar trend was evident when well–moderately differentiated tumours were analysed as a group, but failed to reach statistical significance (4 out of 17 vs 7 out of 11; $P = 0.08$, Fisher's exact test). There was a trend towards increasing AI on 10p and 11p in adenocarcinomas of the moderately differentiated subtype compared with those of the well-differentiated subtype (10p, 7 out of 11 vs 0 out of 5; 11p, 7 out of 9 vs 1 out of 6; both $P < 0.07$, Fisher's exact test) and the poorly differentiated subtype (10p, 7 out of 11 vs 1 out of 8; 11p, 7 out of 9 vs 3 out of 11; both $P < 0.075$, Fisher's exact test). There was no association between AI at a specific chromosomal locus and tumour stage.

To map common regions of involvement on chromosomes 4q and 6q, additional microsatellite markers were analysed on these chromosomes. A number of cases were identified that showed clear evidence of chromosomal breakpoints. Six cases with breakpoints on 4q give preliminary evidence for the involvement of two distinct regions of 4q in gastric tumorigenesis. One region spans an approximate 14 cM interval delineated by retention of heterozygozity at D4S2361 (no. 44) and D4S1647 (no. 37). There is evidence of involvement of a second locus on 4q. Retention of heterozygozity at markers D4S1090 (no. 68) and D4S1652 (no. 98) delineate a maximal 50 cM region of involvement. These data are represented diagrammatically in Figure 3A. Five cases were identified that showed evidence of breakpoints on chromosome 6q. The retention of heterozygozity at D6S1003 (case no. 21) and D6S255 (no. 101) defines a 16-cM interval commonly involved in gastric tumorigenesis (Figure 3B). Examples of AI and retention of heterozygozity at the chromosomal breakpoints are shown in Figure 4.

### DISCUSSION

To identify chromosomal regions implicated in the development of proximal gastric carcinoma, this study presents the results of an allelotype analysis carried out on a series of 38 adenocarcinomas arising in the gastric cardia. The tumour group studied demonstrated a high level of AI for both the intestinal (median FAI = 0.47) and the diffuse (median FAI = 0.54) histological subtypes. These values are higher than those reported in previous studies of gastric carcinoma (Motomura et al, 1988; Wada et al, 1988). This difference may be attributable to a number of factors. First, the tumours in this study consisted only of advanced-stage tumours (stage II or III) with no early-stage tumours (stage 0 or I). Previous studies of ovarian carcinoma have also reported high FAL values in two series of predominantly advanced carcinomas (Cliby et al, 1993). Second, multiple markers were evaluated on most chromosomal arms and on average 35, out of a total of 39, chromosomal arms were informative for each patient. Third, the selection of tumour-rich regions for molecular analysis, may have facilitated a more reliable detection of AI than the study of DNA from unfractionated tumours, a factor that may have contributed to the detection of lower rates of allele loss in some studies (Wada et al, 1988). Finally, adenocarcinomas of the gastric cardia were reported to

© Cancer Research Campaign 1997

**Figure 2** Frequency of allelic imbalance by chromosomal arm, excluding acrocentric arms, detected (**A**) in 29 cases of intestinal-type gastric adenocarcinoma and (**B**) in nine cases of diffuse-type gastric adenocarcinoma. Allelic imbalance for each arm was calculated as the percentage of informative patients showing allelic imbalance of any marker mapping to that arm. The markers analysed in this study are listed in Table 1. ■, p; □, q.

demonstrate higher levels of DNA aneuploidy than those arising in the gastric antrum (Flejou et al, 1994). The majority of tumours in this series exhibited aneuploidy as assessed by flow cytometry [67% (24 out of 36), unpublished data]. The detection of frequent AI at the molecular level may partly reflect this underlying genomic instability. Previous LOH studies of gastric carcinoma have not documented the site of origin of the tumours and it remains to be established if the higher levels of AI documented in this study represent site-specific differences in the aetiology of gastric cancer. The detection of high levels of aneuploidy and AI in proximal gastric carcinomas suggests that inactivation of a key gene controlling genomic stability may be an early and frequent event in these carcinomas.

The present study documented AI on 5q in 58% (22 out of 38) of gastric adenocarcinomas. Allelic loss on 5q has been reported in approximately 30% of gastric carcinomas (Neuman et al, 1991; Sano et al, 1991; McKie et al, 1993; Ranzani et al, 1993; Rhyu et al, 1994). However, loss of the APC/MCC gene loci on 5q was documented in over 80% of flow-sorted, aneuploid cell populations in gastric carcinoma, suggesting that 5q LOH may be associated with the development of aneuploidy in these tumours (Tamura et al, 1993). Inactivation of the APC gene, on 5q21, has been implicated in gastric tumorigenesis by the detection of mutations in 7–21% of tumours (Horii et al, 1992; Nakatsura et al, 1992). The detection of APC gene mutations in gastric adenomas [20% (6 out of 30)] indicates that such mutations may occur early in gastric tumour development (Tamura et al, 1994). Deletion mapping on 5q in well-differentiated gastric adenocarcinoma identified two minimum regions of deletion, both of which were distinct from the APC gene locus (Tamura et al, 1996). These data suggest a role for two additional putative tumour-suppressor genes on 5q in gastric carcinoma.

Microsatellite analysis detected AI on 13q in 53% (20 out of 38) of gastric adenocarcinomas. Motomura et al (1988), reported allelic loss at 13q in 41% (14 out of 34) of gastric adenocarcinomas. However, other studies documented a lower incidence of 13q allelic loss, ranging from 19% to 30% of cases (Uchino et al, 1992; Ranzani et al, 1993; Schneider et al, 1995). The involvement of Rb gene abnormalities in gastric carcinoma has not been reported.

© Cancer Research Campaign 1997

A

| Distance | Location | Locus[a] | Case number | | | | | |
|---|---|---|---|---|---|---|---|---|
| cM | | | 37 | 44 | 94 | 68 | 101 | 98 |
| | 4q | D4S2361 | ▒ | □ | ■ | □ | ▒ | ■ |
| 7 | 4q | D4S2364 | ■ | ▒ | ■ | □ | □ | ■ |
| 7 | 4q21–q23 | D4S1647 | □ | ■ | ■ | ▒ | ▒ | ■ |
| 51 | 4q27–q31 | D4S1625 | □ | □ | ▒ | ▒ | □ | ■ |
| 13 | 4q | D4S1090 | ▒ | ▒ | □ | □ | ■ | ■ |
| 13 | 4q | D4S243 | □ | □ | ▒ | ■ | ■ | ■ |
| 5 | 4q | D4S1646 | ▒ | ▒ | ▒ | ▒ | ■ | ▒ |
| 7.5 | 4q | D4S415 | □ | □ | □ | ■ | ■ | ■ |
| 14 | 4q | D4S408 | □ | □ | □ | ▒ | ■ | □ |
| 5 | 4q33–q35 | D4S171 | ▒ | □ | ▒ | ▒ | ▒ | ▒ |
| 8 | 4q34-qter | D4S1652 | □ | □ | □ | ■ | ▒ | □ |

B

| Distance | Location | Locus[a] | Case number | | | | |
|---|---|---|---|---|---|---|---|
| (cM) | | | 21 | 101 | 105 | 38 | 58 |
| | 6q14–q16.2 | D6S251 | □ | ■ | ■ | □ | □ |
| 36.5 | 6q | D6S474 | ▒ | ■ | ▒ | ▒ | ■ |
| 11 | 6q22–q23 | D6S262 | □ | ■ | ■ | ▒ | ■ |
| 17 | 6q23 | D6S1003 | □ | ▒ | ▒ | ▒ | ■ |
| 9 | 6q | D6S494 | □ | ▒ | ▒ | ■ | ■ |
| 7 | 6q24–q25 | D6S255 | ▒ | □ | ▒ | ■ | ■ |
| 7 | 6q25–q27 | D6S303 | ▒ | ▒ | □ | ■ | ■ |
| 6 | 6q | D6S1035 | □ | □ | □ | ■ | ■ |
| 23.5 | 6q27–qter | D6S503 | □ | □ | □ | ■ | ■ |
| 2.5 | 6q27–qter | D6S1027 | ▒ | ▒ | ▒ | ■ | □ |

**Figure 3** Deletion mapping (**A**) on chromosome 4q, and (**B**) on chromosome 6q. [a] The markers are listed in relative positions from the most centromeric to the most telomeric. ■, Loss of heterozygozity; □, heterozygous; ▒, homozygous/not done

**Figure 4** Representative examples of loss and retention of heterozygosity at breakpoints on 4q and 6q. C, control DNA; T, tumour DNA; AI, allelic imbalance; Het, heterozygous

In this study, the majority of gastric tumours displayed AI at either 17p or 18q or at both loci, suggesting that they are commonly involved in adenocarcinoma of the gastric cardia. Approximately 37.5–74% of gastric carcinomas were reported to demonstrate 17p allelic loss (Sano et al, 1991; Seruca et al, 1992; Ranzani et al, 1993; Rhyu et al, 1994; Schneider et al, 1995). Both p53 gene mutation (Renault et al, 1993; Uchino et al, 1993; Hongyo et al, 1995) and diffuse p53 protein expression (Flejou et al, 1994; Fukunaga et al, 1994) have been documented in gastric tumours, suggesting that p53 gene inactivation is the target of 17p allelic loss in these tumours. Schneider et al (1995) reported low levels of AI at the DCC-linked locus, D18S51 in gastric adenocarcinoma. Other studies, however, have reported levels of 18q LOH ranging from 47% to 61% of tumours, occurring in both early tumours and advanced tumours (Uchino et al, 1992; Ranzani et al, 1993). A putative common region of deletion, including the DCC gene locus, has been identified at 18q21.3–qter (Uchino et al, 1992).

Therefore, the present study and numerous previous reports have implicated allelic loss at 5q, 13q, 17p and 18q in gastric tumorigenesis. These chromosomes harbour the known tumour-suppressor genes APC/MCC, Rb, p53 and DCC (McBride et al, 1986; Lee et al, 1987; Fearon et al, 1990; Kinzler et al, 1991). In addition to these loci, allelic loss has been documented on other chromosomal arms in gastric adenocarcinoma. These include 1q [50% (5 out of 10) Fey et al, 1989; 25% (4 out of 16), Sano et al, 1991], 3p [36% (15 out of 41) Schneider et al, 1995], 7q [(24% (10 out of 41) Sano et al, 1991; 32% (26 out of 82) Kuniyasu et al, 1994], 11p [37% (7 out of 19) Ranzani et al, 1993] and 12q [55% (6 out of 11) Fey et al, 1989; 31% (11 out of 36) Sano et al, 1991; 38% (18 out of 48) Schneider et al, 1995]. This study also detected a number of additional chromosomal loci that exhibited high levels of AI throughout the well-, moderately and poorly differentiated subtypes of intestinal adenocarcinoma and also in diffuse adenocarcinomas. These included 3p, 4q, 6q, 9p, 9q and 12q, and may indicate chromosomal regions containing genes that are involved in the development of adenocarcinoma of the gastric cardia. No associations were observed between AI on a particular chromosomal arm and tumour stage. Weak association trends were documented between AI on 3q, 10p, 11p and 16q, and different histological subtypes of gastric carcinoma.

Ranzani et al (1993) demonstrated frequent LOH at 11p (37%) in gastric adenocarcinoma. Subsequent deletion mapping confirmed a minimum region of deletion at 11p15.5 and also identified a second region of deletion at 11q22–q23 (Baffa et al, 1996). The detection of frequent LOH at 11p15 is consistent with a cytogenetic study detailing 11p13–15 rearrangements in seven out of eight cases of primary adenocarcinoma of the oesophagus and gastric cardia (Rodriguez et al, 1990). In contrast to these observations, other cytogenetic studies failed to detect 11p rearrangements in gastric carcinoma (Ochi et al, 1986; Seruca et al, 1993) and some LOH studies have detected few or no 11p deletions in these

 *© Cancer Research Campaign 1997*

tumours (Sano et al, 1991; Uchino et al, 1992). This study demonstrated AI on 11p in 43% (15 out of 35) of gastric adenocarcinomas, with a trend towards a higher incidence in moderately differentiated tumours (seven out of nine) relative to either well-differentiated (one out of six) or poorly differentiated (3 out of 11) tumours. These data suggest that 11p allelic loss may contribute to the development of a subset of gastric carcinomas.

A detailed molecular analysis of the E-cadherin gene demonstrated gene mutations in 50% (13 out of 26) of diffuse-type carcinomas and 14% (one out of seven) of mixed-type carcinomas (Becker et al, 1994). In contrast, intestinal-type carcinomas demonstrated silent mutations in 2 out of 20 tumours, indicating that E-cadherin gene mutations may contribute to the development of diffusely growing gastric carcinomas (Becker et al, 1994). In agreement with this, the present study detected a trend towards increasing AI on 16q in diffuse-type gastric carcinomas compared with those of the intestinal type ($P = 0.02$, Fisher's exact test). By virtue of its known function and location, E-cadherin represents a potential target of AI on 16q.

Additional studies have suggested that allele loss at a number of loci may be specific to the development of particular subtypes of gastric carcinoma (Sano et al, 1991). One study found evidence to suggest that LOH at 1q, 5q and 7p may be associated with well-differentiated adenocarcinoma, with LOH on 1q and 7p being associated with tumour progression to advanced carcinoma. LOH on these chromosomes was not detected in poorly differentiated adenocarcinoma, including both intestinal and diffuse types (Sano et al, 1991). LOH on 7q was not detected in early tumours, but was evident in advanced tumours of both well- and poorly differentiated subtypes, suggesting an association with tumour progression (Sano et al, 1991; Kuniyasu et al, 1994).

Although Sano et al (1991) reported 5q LOH only in well-differentiated adenocarcinoma, independent studies reported no association between 5q LOH and histological subtype (McKie et al, 1993; Rhyu et al, 1994). Similarly, this study detected AI on 5q in both differentiated and undifferentiated intestinal types of gastric adenocarcinoma and also in the diffuse type. APC gene mutations have been detected more frequently in well-differentiated, intestinal tumours (Nakatsura et al, 1992), but were also reported in undifferentiated and diffuse-type carcinomas, mainly of the signet-ring cell type (Horii et al, 1992; Nakatsura et al, 1992). To date, therefore, studies of gastric carcinoma have documented weak associations (Sano et al, 1991) or no associations (Schneider et al, 1995) between allelic loss/imbalance and tumour stage, histological subtype, or degree of differentiation. In most studies, the small number of tumours in each histological subtype and the predominantly advanced stage of tumour development have precluded the reliable identification of significant associations between allelic loss/imbalance and various clinicopathological parameters. These limitations also apply to the present study, possibly explaining the lack of significant associations between molecular alterations and clinicopathological parameters. These limitations are attributable to the fact that most gastric carcinomas present at an advanced stage (Allum et al, 1989), and that the relatively low incidence of gastric carcinoma in Western countries hampers the acquisition of large tumour series for genetic analysis.

The sites of AI on 4q and 6q were examined in more detail and a subset of tumours was identified that exhibited clear evidence of chromosomal breakpoints. Cytogenetic studies have reported rearrangements involving 4q in both primary (Rodriguez et al, 1990) and metastatic (Cagle et al, 1989) gastric carcinoma. Despite this, one allelotype study failed to detect frequent AI on 4q in gastric carcinoma (Schneider et al, 1995). Allelic loss at 4q has been described relatively infrequently in human malignancies. Buetow et al (1989) reported LOH at the albumin gene locus (4q11–12) in five out of five informative cases of hepatocellular carcinoma. This was confirmed in an independent study by the detection of allelic loss on 4q in 77% (23 out of 30) of hepatocellular carcinomas (Yeh et al, 1996). Allelic loss on 4q has also been implicated in cervical carcinoma (Mitra et al, 1994). The mapping of a senescence function to chromosome 4 provided evidence of a potential tumour-suppressor gene. Introduction of a normal chromosome 4 into three immortal cell lines, derived from a bladder carcinoma, a cervical carcinoma and a glioblastoma, was shown to result in loss of proliferation and reversal of the immortal phenotype (Ning et al, 1991).

A number of cytogenetic studies have reported consistent abnormalities involving 6q21–6qter in gastric carcinoma (Ochi et al, 1986; Rodriguez et al, 1990; Seruca et al, 1993; Panani et al, 1995; Rao et al, 1995), with one study documenting five tumours with a commonly deleted region spanning 6q21–6qter (Seruca et al, 1993). A number of LOH studies failed to detect high levels of 6q allelic loss in gastric adenocarcinoma (Sano et al, 1991; Uchino et al, 1992). More recently, deletion mapping on 6q in gastric carcinoma has identified two regions of deletion; one located between D6S268 (6q16–q21) and ARG1 (6q22–q23), and a second region distal to IFNGR1 (6q23–q24) (Queimado et al, 1995). It will be of interest to determine if the minimum region identified in the present study, located between D6S1003 (6q23) and D6S255 (6q24–q25), corresponds to one of those identified in the other mapping study. Evidence in the literature suggests the presence of a number of tumour-suppressor genes on chromosome 6q. Frequent 6q LOH has been described in a variety of tumour types, including breast (Orphanos et al, 1995), ovarian (Cliby et al, 1993) and colorectal (Honchel et al, 1996) carcinoma. Microcell-mediated transfer of normal chromosome 6 was reported to result in reversion of the malignant phenotype in melanoma cell lines (Trent et al, 1990). In agreement with the presence of a tumour-suppressor gene on 6q, loss of chromosomal loci distal to 6q21 was associated with the immortalization of SV40-transformed human diploid fibroblasts (Hubbard-Smith et al, 1992).

In conclusion, this study documents the pattern of AI in a series of proximally located gastric adenocarcinomas. Gastric carcinoma is a heterogeneous disease, with proximal and distal gastric tumours displaying distinct biological and epidemiological features (Wang et al, 1986; Blot et al, 1991). To date, LOH studies in gastric carcinoma have reported diverse patterns of allele loss and accurate classification with respect to site of origin, tumour morphology and stage is vital to the elucidation of the genetic mechanisms underlying distinct subtypes of gastric cancer. This study provides the basis for a more detailed molecular analysis of proximal gastric adenocarcinoma.

### ACKNOWLEDGEMENTS

This work was supported by a grant from the Northern Ireland Chest, Heart and Stroke Association and NIH Grant HG00835.

### ABBREVIATIONS

LOH, loss of heterozygozity, AI, allelic imbalance; PCR, polymerase chain reaction; FAI, fractional allelic imbalance.

© Cancer Research Campaign 1997

### REFERENCES

Allum WH, Powell DJ, McConkey CC and Fielding JWL (1989) Gastric cancer: a 25-year review. *Br J Surg*, **76**: 535–540

Baffa R, Negrini M, Mandes B, Rugge M, Ranzani G, Hirohashi S and Croce CM, (1996), Loss of heterozygosity for chromosome 11 in adenocarcinoma of the stomach. *Cancer Res* **56**: 268–272

Baker SJ, Preisinger AC, Jessup JM, Paraskeva C, Markowitz S, Willson JKV, Hamilton S and Vogelstein B (1990), p53 gene mutations occur in combination with 17p allelic deletions as late events in colorectal tumorigenesis. *Cancer Res* **50**: 7717–7722

Beahrs OH, Henson D, Hutter RVP and Myers MH (1988) *Manual for Staging Cancer, American Joint Committee on Cancer*. JB Lippincott: Philadelphia

Becker K-F, Atkinson MJ, Reich U, Becker I, Nekarda H, Siewert JR and Hofler H (1994) E-cadherin gene mutations provide clues to diffuse type gastric carcinomas. *Cancer Res* **54**: 3845–3852

Blot WJ, Devesa SS, Kneller RW and Fraumeni JF (1991) Rising incidence of adenocarcinoma of the esophagus and gastric cardia. *JAMA* **265**: 1287–1289

Buetow KH, Murray JC, Israel JL, London WT, Smith M, Klew M, Blanquet V, Brechot C, Redeker A and Govindarajah S (1989) Loss of heterozygosity suggests tumor suppressor gene responsible for primary hepatocellular carcinoma. *Proc Natl Acad Sci USA* **86**: 8852–8856

Cagle PT, Taylor LD, Schwartz MR, Ramzy I and Elder FFB (1989) Cytogenetic abnormalities common to adenocarcinoma metastatic to the pleura. *Cancer Genet Cytogenet* **39**: 219–225

Cliby W, Ritland S, Hartmann L, Dodson M, Halling KC, Keeney G, Podratz KC and Jenkins RB (1993) Human epithelial ovarian cancer allelotype. *Cancer Res* **53**: 2393–2398

Fearon ER, Cho KR, Nigro JM, Kern SE, Simons JW, Ruppert JM, Hamilton SR, Preisinger AC, Thomas G, Kinzler KW and Vogelstein B (1990) Identification of a chromosome 18q gene that is altered in colorectal cancers. *Science* **247**: 49–56

Ferti-Passantonopoulou AD, Panani AD, Vlachos JD and Raptis SA (1987) Common cytogenetic findings in gastric cancer. *Cancer Genet Cytogenet* **24**: 63–73

Fey MF, Hesketh C, Wainscoat JS, Gendler S and Thein SL (1989) Clonal allele loss in gastrointestinal cancers. *Br J Cancer* **59**: 750–754

Flejou J-F, Muzeau F, Potet F, Lepelletier F, Fekete F and Henin D (1994) Overexpression of the p53 tumor suppressor gene product in esophageal and gastric carcinomas. *Path Res Pract* **190**: 1141–1148

Fukunaga M, Monden T, Nakanishi H, Ohue M, Fukuda K, Tomita N, Shimano T and Mori T (1994) Immunohistochemical study of p53 in gastric carcinoma. *Am J Clin Pathol* **101**: 177–180

Gleeson CM, Sloan JM, McGuigan JA, Ritchie AJ, Weber JL, Russell SEH (1996) Widespread microsatellite instability occurs infrequently in adenocarcinoma of the gastric cardia. *Oncogene* **12**: 1653–1662

Hartwell L (1992) Defects in a cell cycle checkpoint may be responsible for the genomic instability of cancer cells. *Cell* **71**: 543–546

Hermanek P and Sobin LH (1987) *Classification of Malignant Tumors*. Springer Berlin

Honchel R, McDonnell S, Schaid DJ and Thibodeau SN (1996) Tumor necrosis factor alpha allelic frequency and chromosome 6 allelic imbalance in patients with colorectal cancer. *Cancer Res* **56**: 145–149

Hongyo T, Buzard GS, Palli D, Weghorst CM, Amorosi A, Galli M, Caporaso NE, Fraumeni Jr JF and Rice JM (1995) Mutations of the K-*ras* and *p53* genes in gastric adenocarcinomas from a high-incidence region around Florence, Italy. *Cancer Res* **55**: 2665- 2672

Horii A, Nakatsuru S, Miyoshi Y, Ichii S, Nagase H, Kato Y, Yanagisawa A and Nakamura Y (1992) The *APC* gene, responsible for familial adenomatous polyposis, is mutated in human gastric cancer. *Cancer Res* **52**: 3231–3233

Hubbard-Smith K, Patsalis P, Pardinas JR, Jha KK, Henderson AS and Ozer HL (1992) Altered chromosome 6 in immortal human fibroblasts. *Mol Cell Biol* **12**: 2273–2281

Kinzler KW, Nilbert MC, Su L-K, Vogelstein B, Bryan TM, Levy DB, Smith KJ, Preisinger AC, Hedge P, McKechnie D, Finniear R, Markham A, Groffen J, Boguski MS, Altschul SF, Horii A, Ando H, Miyoshi Y, Miki Y, Nishisho I and Nakamura Y (1991) Identification of FAP locus genes from chromosome 5q21. *Science* **253**: 661–665

Kuniyasu H, Yasui W, Yokozaki H, Akagi M, Akama Y, Kitahara K, Fujii K and Tahara E (1994) Frequent loss of heterozygosity of the long arm of chromosome 7 is closely associated with progression of human gastric carcinomas. *Int J Cancer* **59**: 597–600

Lauren P (1965) The two histological main types of gastric adenocarcinoma: diffuse and so called intestinal-type adenocarcinoma. *Acta Pathol Microbiol Immunol Scand* **5**: 145–153

Lee W-H, Bookstein R, Hong F, Young L-J, Shew J-Y and Lee EY-HP (1987) Human retinoblastoma susceptibility gene: Cloning, identification, and sequence. *Science* **235**: 1394–1399

McBride OW, Merry D and Givol D (1986) The gene for human p53 cellular tumor antigen is located on chromosome 17 short arm (17p13). *Proc Natl Acad Sci USA* **83**: 130–134

McKie AB, Filipe MI and Lemoine NR (1993) Abnormalities affecting the APC and MCC tumour suppressor gene loci on chromosome 5q occur frequently in gastric cancer but not in pancreatic cancer. *Int J Cancer* **55**: 598–603

Mitra AB, Murty VS, Li RG, Pratap M, Luthra UK and Chaganti RSK (1994) Allelotype analysis of cervical carcinoma. *Cancer Res* **54**: 4481–4487

Motomura K, Nishisho I, Takai S-I, Tateishi H, Okazaki M, Yamamoto M, Miki T, Honjo T and Mori T (1988) Loss of alleles at loci on chromosome 13 in human primary gastric cancers. *Genomics* **2**: 180–184

Nakatsura S, Yanagisawa A, Ichii S, Tahara E, Kato Y, Nakamura Y and Horii A (1992) Somatic mutation of the *APC* gene in gastric cancer: frequent mutations in very well differentiated adenocarcinoma and signet-ring cell carcinoma. *Hum Mol Genet* **1**: 559–563

Neuman WL, Wasylyshyn ML, Jacoby R, Erroi F, Angriman I, Montag A, Brasitus T, Michelassi F and Westbrook CA (1991) Evidence for a common molecular pathogenesis in colorectal, gastric, and pancreatic cancer. *Genes Chromosom Cancer* **3**: 468–473

Ning Y, Weber JL, Killary AM, Ledbetter DH, Smith JR and Pereira-Smith OM (1991) Genetic analysis of indefinite division in human cells: Evidence for a cell senescence-related gene(s) on human chromosome 4. *Proc Natl Acad Sci USA* **88**: 5635–5639

Nowell PC (1976) The clonal evolution of tumor cell populations. *Science* **194**: 23–28

Ochi H, Douglass JHO and Sandberg AA (1986) Cytogenetic studies in primary gastric cancer. *Cancer Genet Cytogenet* **22**: 295–307

Orphanos V, McGown G, Hey Y, Boyle JM and Santibanez-Koref M (1995) Proximal 6q, a region showing allele loss in primary breast cancer. *Br J Cancer* **71**: 290–293

Panani AD, Ferti A, Malliaros S and Raptis S (1995) Cytogenetic study of 11 gastric adenocarcinomas. *Cancer Genet Cytogenet* **81**: 169–172

Powell SM, Zilz N, Beazer-Barclay Y, Bryan TM, Hamilton SR, Thibodeau SN, Vogelstein B and Kinzler KW (1992) APC mutations occur early during colorectal tumorigenesis. *Nature* **359**: 235–237

Queimado L, Seruca R, Costa-Pereira A and Castedo S (1995) Identification of two distinct regions of deletion at 6q in gastric carcinoma. *Genes Chromosom Cancer* **14**: 28–34

Ranzani GN, Renault B, Pellegata NS, Fattorini P, Magni E, Bacci F and Amadori D (1993) Loss of heterozygosity and K-*ras* gene mutations in gastric cancer. *Hum Genet* **92**: 244–249

Rao PH, Mathew S, Kelsen DP and Chaganti RS (1995) Cytogenetics of gastric and esophageal adenocarcinomas. 3q deletion as a possible primary chromosomal change. *Cancer Genet Cytogenet* **81**: 139–143

Renault B, Van Den Broek M, Fodde R, Wijnen J, Pellegata NS, Amadori D, Khan PM and Ranzani GN (1993) Base transitions are the most frequent changes at p53 in gastric cancer. *Cancer Res* **53**: 2614–2617

Rhyu M-G, Park W-S, Jung Y-J, Choi S-W and Meltzer SJ (1994) Allelic deletions of *MCC/APC* and *p53* are frequent late events in human gastric carcinogenesis. *Gastroenterology* **106**: 1584–1588

Rodriguez E, Rao PH, Ladanyi M, Altorki N, Albino AP, Kelsen DP, Jhanwar SC and Chaganti RSK (1990) 11p13-15 is a specific region of chromosomal rearrangement in gastric and esophageal adenocarcinomas. *Cancer Res* **50**: 6410–6416

Sano T, Tsujino T, Yoshida K, Nakayama H, Haruma K, Ito H, Nakamura Y, Kajiyama G and Tahara E (1991) Frequent loss of heterozygosity on chromosomes 1q, 5q, and 17p in human gastric carcinomas. *Cancer Res* **51**: 2926–2931

Schneider BG, Pulitzer DR, Brown RD, Prihoda TJ, Bostwick TJ, Bostwick DG, Saldivar V, Rodriguez-Martinez HA, Gutierrez-Diaz ME and O'Connell P (1995) Allelic imbalance in gastric cancer: An affected site on chromosome arm 3p. *Genes Chromosom Cancer* **13**: 263–271

Seruca R, David L, Holm R, Nesland JM, Fangan BM, Castedo S, Sobrinho-Simoes M and Borresen A-L (1992) p53 mutations in gastric carcinomas. *Br J Cancer* **65**: 708–710

Seruca R, Castedo S, Correia C, Gomes P, Carneiro F, Soares P, De Jong B and Sobrinho-Simoes M (1993) Cytogenetic findings in eleven gastric carcinomas. *Cancer Genet Cytogenet* **68**: 42–48

Sidoni A, Lancia D, Pietropaoli N and Ferri I (1989) Changing patterns in gastric carcinoma. *Tumori* **75**: 605–608

© Cancer Research Campaign 1997

Tamura G, Maesawa C, Suzuki Y, Ogasawara S, Terashima M, Saito K and Satodate R (1993) Primary gastric carcinoma cells frequently lose heterozygosity at the APC and MCC genetic loci. *Jpn J Cancer Res* **84**: 1015–1018

Tamura G, Maesawa C, Suzuki Y, Tamada H, Satoh M, Ogasawara S, Kashiwaba M and Satodate R (1994) Mutations of the *APC* gene occur during early stages of gastric adenoma development. *Cancer Res* **54**: 1149–1151

Tamura G, Ogasawara S, Nishizuka S, Sakata K, Maesawa C, Suzuki Y, Terashima M, Saito K and Satodate R (1996) Two distinct regions of deletion on the long arm of chromosome 5 in differentiated adenocarcinomas of the stomach. *Cancer Res* **56**: 612–615

Trent JM, Stanbridge EJ, McBride HL, Meese EU, Casey G, Araujo DE, Witkowski CM and Nagle RB (1990) Tumorigenicity in human melanoma cell lines controlled by introduction of human chromosome 6. *Science* **247**: 568–571

Uchino S, Tsuda H, Noguchi M, Yokota J, Terada M, Saito T, Kobayashi M, Sugimura T and Hirohashi S (1992) Frequent loss of heterozygosity at the DCC locus in gastric cancer. *Cancer Res* **52**: 3099–3102

Uchino S, Noguchi M, Ochiai A, Saito T, Kobayashi M and Hirohashi S (1993) p53 mutation in gastric cancer: a genetic model for carcinogenesis is common to gastric and colorectal cancer. *Int J Cancer* **54**: 759–764

Vogelstein B, Fearon ER, Hamilton SR, Kern SE, Preisinger AC, Leppert M, Nakamura Y, White R, Smits AMM and Bos JL (1988) Genetic alterations during colorectal-tumor development. *N Engl J Med* **319**: 525–532

Vogelstein B, Fearon ER, Kern SE, Hamilton SR, Preisinger AC, Nakamura Y and White R (1989) Allelotype of colorectal carcinomas. *Science* **244**: 207–211

Wada M, Yokota J, Mizoguchi H, Sugimura T and Terada M (1988) Infrequent loss of chromosomal heterozygosity in human stomach cancer. *Cancer Res* **48**: 2988–2992

Wang HH, Antonioli DA and Goldman H (1986) Comparative features of esophageal and gastric adenocarcinomas: Recent changes in type and frequency. *Hum Pathol* **17**: 482–487

Watanabe H, Jass JR and Sobin LH (1990) *Histologic Typing of Gastric and Oesophageal Tumors. WHO International Classification of Tumors*. Springer: Berlin

Yeh S-H, Chen M-Y, Lai M-Y and Chen D-S (1996) Allelic loss on chromosomes 4q and 16q in hepatocellular carcinoma: Association with elevated alpha-fetoprotein production. *Gastroenterology* **110**: 184–192

© Cancer Research Campaign 1997